# Experimental investigation of the machining characteristics in diamond wire sawing of unidirectional CFRP

**DOI:** 10.1007/s00170-021-07146-8

**Published:** 2021-06-09

**Authors:** Lukas Seeholzer, Stefan Süssmaier, Fabian Kneubühler, Konrad Wegener

**Affiliations:** Institute of Machine Tools and Manufacturing (IWF), ETH, Zürich, Switzerland

**Keywords:** CFRP, Fibre-reinforced plastic, Diamond wire sawing, Machining

## Abstract

Especially for slicing hard and brittle materials, wire sawing with electroplated diamond wires is widely used since it combines a high surface quality with a minimum kerf loss. Furthermore, it allows a high productivity by machining multiple workpieces simultaneously. During the machining operation, the wire/workpiece interaction and thus the material removal conditions with the resulting workpiece quality are determined by the material properties and the process and tool parameters. However, applied to machining of carbon fibre reinforced polymers (CFRP), the process complexity potentially increases due to the anisotropic material properties, the elastic spring back potential of the material, and the distinct mechanical wear due to the highly abrasive carbon fibres. Therefore, this experimental study analyses different combinations of influencing factors with respect to process forces, workpiece surface temperatures at the wire entrance, and the surface quality in wire sawing unidirectional CFRP material. As main influencing factors, the cutting and feed speeds, the density of diamond grains on the wire, the workpiece thickness, and the fibre orientation of the CFRP material are analysed and discussed. For the tested parameter settings, it is found that while the influence of the grain density is negligible, workpiece thickness, cutting and feed speeds affect the process substantially. In addition, higher process forces and workpiece surface temperatures do not necessarily deteriorate the surface quality.

## Introduction

Carbon fibre reinforced polymers (CFRP) are characterised by high specific strength and stiffness properties that make them particularly suitable for lightweight constructions found in aerospace and automotive applications [4, 8]. Although CFRP components are usually produced near net shape, additional machining operations are necessary for finishing as stated by Sheikh-Ahmad [22]. According to Geier et al. [5], hole drilling and edge trimming are the most important finishing operations. While holes are usually produced by conventional drilling, different machining strategies are used for trimming of CFRP.

In this context, Negarestani and Li [14] mention mechanical machining with geometrically defined cutting edge, wire electric discharge machining (WEDM), abrasive water jet machining (AWJM), and laser cutting as the most common trimming technologies. As explained in the following, each of these methods is characterised by process-specific advantages and disadvantages.

According to Rummenhöller [20], mechanical machining of CFRP with geometrically defined cutting edge is challenging due to the material’s anisotropy and heterogeneity resulting in fibre orientation–dependent cutting mechanisms and surface qualities. Furthermore, numerous experimental studies [15, 21, 23] showed that machining CFRP is associated to extensive mechanical tool wear because of the highly abrasive carbon fibres. According to Voss et al. [26], progressive tool wear usually results in an increasing cutting edge radius and an increasing friction length on the flank face due to a decreasing clearance angle. In consequence, the cutting forces and temperatures increase as the tool/material interaction changes, which, in accordance with Wang et al. [28], causes a higher risk for process-related damages, e.g. delamination, fibre pull-outs, matrix burning, and uncut fibres. Although tool performance and tool life can be increased by using diamond or diamond-like coatings and specific tool geometries, the machining quality has to be monitored and manual rework is often necessary.

Abrasive waterjet is a well-established method for trimming of CFRP. According to Van Luttervelt [25], the process-related advantages are the relatively small cutting forces and the absence of thermal distortion in the cutting zone. Since material removal is realised by abrasive particles in the water jet, this trimming method is free of tool wear, except for wear in the mixing chamber and the nozzle. Most experimental studies identified kerf loss as a critical issue in AWJM, which is due to the interaction between the water jet and the material. According to Caydas and Hascalik [2], the typical cutting zone produced by AWJM can be separated into three regions, i.e. the initial damage region (IDR), the smooth cutting region (SCR), and the rough cutting region (RCR). The IDR describes the entrance zone of the water jet, where the erosion of the abrasive particles causes an edge rounding, which leads to an unwanted widening of the cutting width as shown by Wang [27]. With increasing penetration depth, the water jet stabilises and allows a more uniform cut in the SCR. However, with increasing penetration depth, the kinetic energy of the water jet decreases and hence the associated cutting capacity is reduced. Especially in the RCR close to the water jet exit, this results in a reduction of the cutting width and a rough cutting surface [1]. According to Monoranu et al. [12], the formation of these three distinctive zones is responsible for the kerf loss mentioned above. As stated by Yang et al. [31], the kerf loss can be reduced by using optimised process parameters; however, it cannot be removed completely. According to Phapale et al. [18], the risk of delamination increases with a higher water pressure, a higher abrasive-mass flow rate and an increased stand-off distance.

Since carbon fibres, unlike glass or aramid fibres, are good electrical conductors, WEDM can be used for trimming of CFRP as shown by Lau et al. [10]. In contrast to most of the remaining trimming methods, WEDM allows the formation of curved edge profiles by controlling the upper and lower wire guides separately [25]. Furthermore, Negarestani [13] mentions the good cutting edge quality and surface finish as further process advantages of WEDM in machining of CFRP. In return, Negarestani and Li [14] emphasise the risk of thermal-related workpiece damages during the machining process, the comparable low material removal rate, and the high investment costs for the infrastructure as critical drawbacks of WEDM.

As shown by different experimental studies [8, 19, 32], laser cutting can be used as an alternative trimming method. According to Herzog et al. [8], this machining method combines a high reliability due the absence of wear with high process flexibility and high cutting speeds. However, since the material removal is realised by thermal ablation, laser cutting is associated to a high risk for thermal damages within the heat-affected zone (HAZ) [24]. Accordingly, numerous researchers have focused on minimising the HAZ by optimising the laser parameters and the laser path control. As stated by Riveiro et al. [19], the physical properties and thus the material’s reaction on induced thermal energy are significantly different for the matrix and the fibre. In consequence, this often results in an insufficient surface integrity due to matrix recession, matrix decomposition, and/or delamination. Furthermore, according to Herzog et al. [8], the maximum material thickness that can be cut by laser is limited due to the caustic of the laser beam.

Especially for slicing hard and brittle materials, i.e. silicon, wire sawing with multi-wire saws is a well-established and widely used manufacturing technology. According to Kumar and Melkote [9], this is because of the process-specific high productivity and the good surface quality, as well as the low kerf loss. The process uses a moving tensioned wire with abrasives as a tool. The wire travels at high speeds along its axis and in feed direction against a workpiece resulting in a mechanical material removal. In recent years, the wire sawing process using wires with fixed diamonds abrasives is replacing a slurry-based process, in which the moving wire pulls abrasives into the kerf to constitute a lapping process. Wrapping wire around guiding rolls to form a thinly spaced web allows for cutting of several hundred wafers at once on multi-wire saws. This justifies the industrial significance and explains why the wafer production costs have decreased notably. Compared to multi-wire saws, single wire saws are less bound to a very specific application and thus offer a wider field of application. A prominent application of a single wire is the cropping of drawn crystals to transform a round workpiece into a square ingot with flat ends. Some industrial reports commend the flexibility of single wire saws to produce precise separation and 2D cuts without significant heat affection in different materials such as glass, ceramics, polymers, and metals, but especially composite materials.

While the most popular trimming methods represented by machining with geometrically defined cutting edge and AWJM are extensively investigated by the research community, only little research work exists for wire sawing of CFRP. In this context, the most comprehensive experimental study was performed by Zhang and Tani [33] in 2017. The authors cut unidirectional (UD) CRFP plates with a fibre content of 67 vol. − *%* with electroplated diamond wires of 0.15mm diameter. As typically done on open wire machines, the cutting direction was reversed periodically, resulting in an intermittent cut where the wire has to be accelerated and decelerated and cutting speeds are limited by the acceleration of the machine and the wire length. Cutting speeds of *v*_*c*_ = 3 − 10 m/s and feed speeds of 2.5–10 mm/min were evaluated for cutting fibres vertically (corresponding to a fibre orientation *𝜃* = 0^∘^ in the denotation used in this study). While no quantifiable results were presented, it was noted that the chip size and the roughness increase with the feed speed. A dependency of the cutting and feed forces on the wire tension was observed which, in accordance with previous work performed by Liedke and Kuna [11], can be explained by the relationship between the wire bow formation and the process parameters.

Although only little research is focused on wire sawing of CFRP, this trimming method combines some unique process advantages if compared to the alternative machining method explained above. These are the low kerf loss combined with negligible heat input during machining resulting in high quality cuts with respect to form accuracy and surface quality. Especially in applications, where high-quality standards are required, thermally induced material property changes leading to reduced material strength as shown by Herzog et al. [7], are not acceptable. Therefore, WEDM and laser cutting are not suitable due to their material removal mechanisms leaving only AWJM and mechanical machining with geometrically defined cutting edge as promising alternatives. However and as mentioned before, AWJM has the characteristics of nonuniform cuts affecting the quality of the cut with respect to form and surface finish. Similarly, cutters are exposed to extensive and complex tool wear, which affects the tool performance with increasing cutting length in terms of the resulting machining quality and the process reliability as highlighted by Hashish et al. [6]. Additional rework and an increasing risk of material weakening, for instance due to delamination, are significant cost drivers.

In this experimental study, wire sawing with diamond grains as fixed abrasives is used to cut UD CFRP material. In this context, machining experiments are performed for different fibre orientations, grain densities, material thicknesses, and cutting and feed speeds. While the machining conditions are quantified by the process forces and the process temperatures, the machining quality is linked to the surface quality in terms of roughness and waviness.

## Materials and methods

In this section, the experimental setup, the workpiece material, and the evaluation methods applied are detailed.

### Experimental setup

**Fig. 1:**
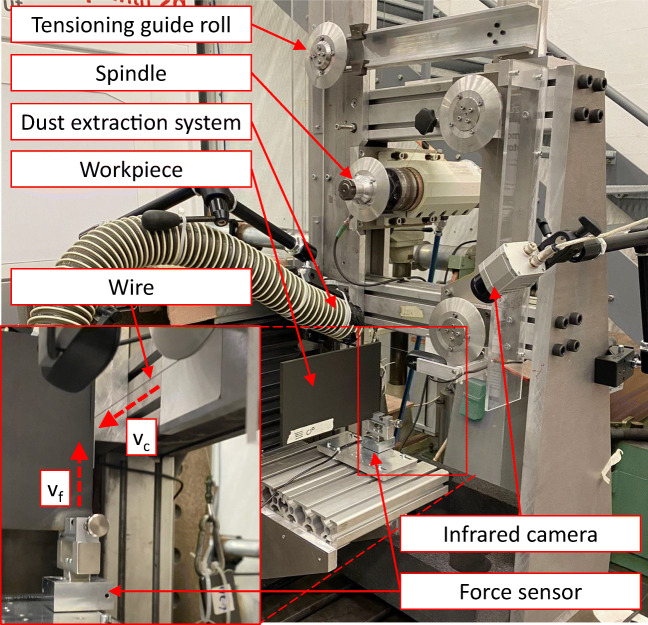
Diamond wire saw used for the cutting experiments

**Fig. 2:**
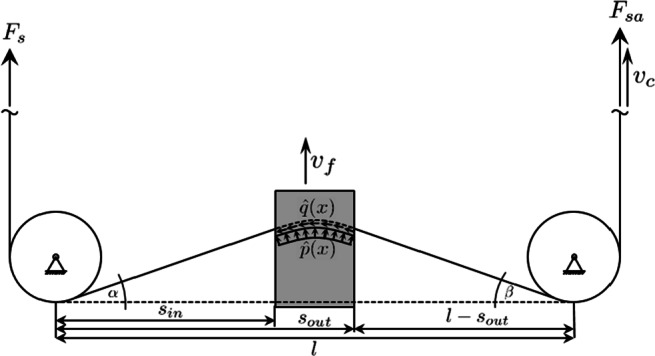
Kinematics of the diamond wire sawing process

The experiments are conducted on a self-build diamond wire saw using a single wire loop. The machine is shown in Fig. 1. The motions and acting forces are visualised in Fig. 2.

A 1.95m long wire loop is led around five guide rolls, where one roll is driven by a spindle and another one is hinged for wire tension control. The workpiece is fed with a constant feed speed *v*_*f*_ into the moving wire between the two bottom guides. The tension in the wire *F*_*s*_ counteracts the wire displacement, exerting a distributed normal force $\hat p(x)$ onto the wire. For small wire bow angles *α* and *β* and a centred placement of the workpiece between the two guide rolls, this force equals approximately the feed force *F*_*f*_ captured by a strain-gauge based force sensor (ME K3D40-20N connected to a GSV4-USB bridge amplifier and analogue-digital-converter), which is mounted in between the workpiece and the linear feed axis. The wire moves in uniform direction with cutting speed *v*_*c*_, removing material and exerting a longitudinally distributed cutting force distribution $\hat q(x)$, which under the same assumptions as before can be measured as the cutting force *F*_*c*_ below the workpiece. The cutting force leads to a larger wire tension *F*_*sa*_ on the wire exit side.

An infrared camera type Optris PI 640 is used to measure the process temperatures on the workpiece surface on the wire entrance side. This camera allows thermal measurements between –20 ^∘^C and 900 ^∘^C with a maximum frame rate of 125 Hz. Based on the black surface of the CFRP material, the emissivity factor is chosen to be 1.

### Tool: Wire

**Fig. 3:**
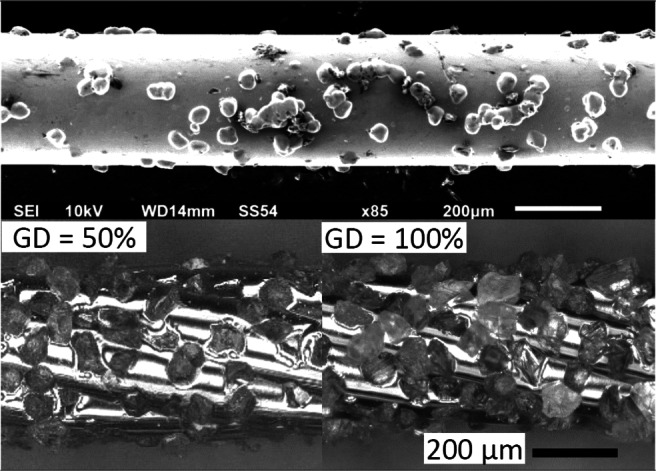
Diamond wire-top: standard electroplated wire as used for cutting silicon, bottom: wires used in this study with 50*%* (left) and 100*%* (right) grain density

Two different wires are used in this study, type EL-MS-045D-50 with 50*%* and type EL-MS045D-100 with 100*%* grain density respectively supplied by INSOLL Tools Technology. Due to the requirement of being connected to a single loop, these wires differ from the electroplated diamond wires used on industrial wire saws for slicing hard and brittle materials where the wire is typically unwound. A comparison of the two wires used with a typical electroplated wire is shown in Fig. 3.

A typical electroplated diamond wire consists of a steel core with diamonds fixed with a filler layer made from a nickel or nickel-cobalt alloy. Typical core diameters lie in the range of 60–140 *μm* with grain sizes between 8–25 *μm*. INSOLL wires are stranded wires coated with an unspecified metal layer where the diamonds are pressed into. The diamonds lay bare and the grain density is much higher compared to electroplated wires as exemplarily shown in Fig. 3. The core diameter of the strand is 450 *μm* and the grain sizes are in the range of 50–100 *μm*.

### CRFP material

**Fig. 4:**
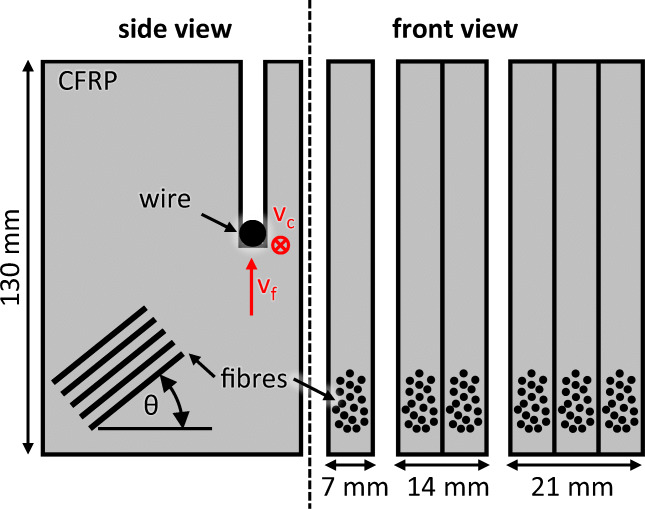
Schematic illustration of the machining situation with standard and stacked CFRP plates

For the experiments, the UD CFRP sheet material type MTM44-1/HTS(12K)-134-35%RW is used, which is characterised by one identical fibre orientation through all laminate layers. This material contains the high performance epoxy matrix type MTM44-1 and the high strength aerospace grade carbon fibres type HTS, which are arranged to rovings of 12,000 fibres each. Some important mechanical properties of the CFRP material are summarised in Table 1. In total, four different fibre orientations are tested, namely *𝜃* = 0^∘^, *𝜃* = 30^∘^, *𝜃* = 60^∘^, and *𝜃* = 90^∘^. As schematically shown in Fig. 4, the fibre orientation angle *𝜃* is measured clockwise from the fibre axis to the horizontal edge of the workpiece, which is perpendicular to the feed direction.

**Table 1:** Mechanical properties of CFRP material

Fibre volume (by weight) [*%*]	65
Laminate density [*g*/cm^3^]	1.35
Tensile strength* [*MPa*]	2159
Tensile modulus [*GPa*]	128.9
Compression strength** [*MPa*]	1330
Compression modulus [*GPa*]	123.3

*ASTM D3039, **ASTM D3410

For the experiments, the CFRP material is prepared in the dimensions 200 mm × 130 mm × 7 mm, where the material thickness corresponds to the longitudinal contact length of the wire. As explained in detail in Section 2.4, some experiments are performed with larger material thicknesses of 14 mm and 21 mm. This is realised by stacking two and three standard plates respectively. No binder is used for stacked material plates; instead, they are clamped mechanically. The analysed feed path per repetition is *l*_*feed*_ = 130 mm, which is identical to the width of the CFRP material.

### Processing parameters

Some considerations are taken into account regarding the choice of process parameters and its ranges. The grain density *GD* is tested on two settings to estimate whether the unconventionally high grain density has a significant effect on the process behaviour. From mechanical machining, it is known that the fibre cutting angle has a major influence with respect to the fibre failure mechanism as described in Section 1, which is why the fibre orientation *𝜃* is tested in this study. Two experiments with a larger material thickness *t* are performed since limitations of other cutting methods become more evident with increasing cutting depth. In order to show the economic potential of the wire cutting, the feed speed as well as the cutting speed are varied in the scope of this work. Lower range cutting and feed speeds have been analysed by Zhang and Tani [33]. However, the higher speed range increases productivity by exploiting the capability of the test rig and allows for the analysis of potential drawbacks such as excessive heat generation.

The experimental plan used is show in Table 2. Wire tension is kept constant at 25 N and in combination with a guide roll distance of 300 mm, a maximum wire deflection angle of 3.5^∘^ is observed for setting 19. Process forces and surface temperatures are measured during the experiments. Each setting is repeated three times with the exception of the settings 13 to 16, which are conducted only once. Experiments with a grain density of 50*%* are performed first before the experiments with a grain density of 100*%*. The order of the experiments and repetitions is randomised apart from settings 18 and 19. All repetitions of setting 18 are executed before setting 19 and both are performed at the end of the runs with a grain density of 50*%*.

**Table 2:** Experimental plan

*ID*	*𝜃* [^∘^]	*GD* [*%*]	*t* [*mm*]	*v*_*f*_ [$\frac {mm}{min}$]	*v*_*c*_ [$\frac {m}{s}$]
1	0	50	7	100	25
2	30	50	7	100	25
3	60	50	7	100	25
4	90	50	7	100	25
5	0	50	7	200	25
6	30	50	7	200	25
7	60	50	7	200	25
8	90	50	7	200	25
9	0	100	7	100	25
10	30	100	7	100	25
11	60	100	7	100	25
12	90	100	7	100	25
13	0	100	7	200	25
14	30	100	7	200	25
15	60	100	7	200	25
16	90	100	7	200	25
17	0	50	7	100	50
18	0	50	14	100	25
19	0	50	21	100	25

### Analysis

The process results are quantified in terms of the feed force *F*_*f*_, the cutting force *F*_*c*_, the workpiece surface temperature *T*, and the topography parameter mean roughness and waviness *R*_*a*_, *W*_*a*_ and peak-to-valley roughness and waviness *R*_*z*_, *W*_*z*_. Some considerations and assumptions are made for the determination of the values, which are explained in the following paragraphs.

#### Process forces:

**Fig. 5:**
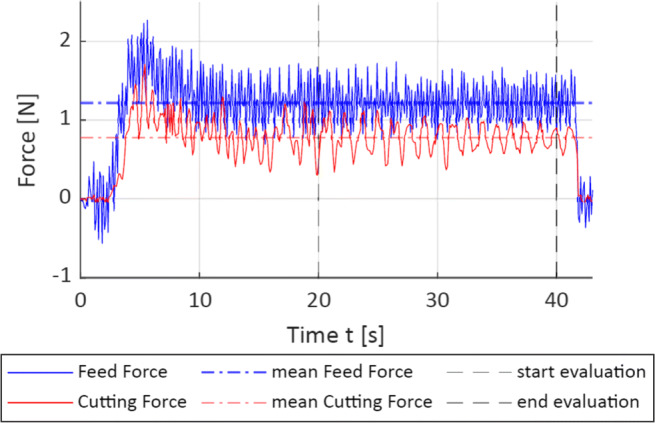
Example of a measured force signal, parameter setting 6 in Table 2, with vertical lines indicating the beginning and the end of the section evaluated for the determination of mean forces

Process forces are recorded with a low sample rate of 20 Hz. This implies that the analysis of dynamic forces, e.g. vibrations or rapid changes, with a frequency higher than 10 Hz is not possible. When vibration is disregarded, the dynamics of the wire sawing process is very low. As the workpiece is pressed against the compliant wire, a bow develops until the reactive force stemming from the wire tension opposes the feed in such a way that the resultant material removal rate equals to the feed rate. The steady state force considered for all performed experiments is reached at the latest after 15 s. From that equilibrium point onward, the process is regarded as stationary, the wire bow, and the process forces do not vary and remain on a nearly constant value. Changes in the removal rate, for instance due to wire wear or clogging, may lead to a slow rise in process forces, which, however, is not observed in this study. Exemplary force signals for the cutting and feed forces are shown in Fig. 5. In order to derive mean force values, the signals for feed and cutting forces are averaged over the last 20 s of the cuts, corresponding approximately to the last 30 mm for the settings with *v*_*f*_ = 100 mm/min and 60 mm for *v*_*f*_ = 200 mm/min respectively.

#### Workpiece temperature:

**Fig. 6:**
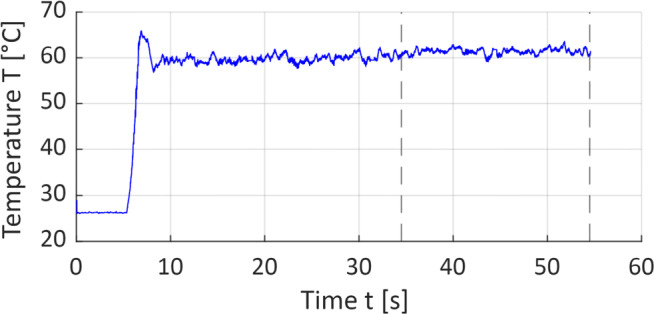
Example of a temperature signal, parameter setting 2 in Table 2, with vertical lines indicating the beginning and the end of the section evaluated for the determination of mean workpiece temperature; run-in period is visible as a peak in the beginning of the signal

For the temperature measurements, a frame rate of 125 Hz and a measuring range of 0–100 ^∘^C is used. The thermal camera is oriented perpendicular to the workpiece surface at the wire entrance and the maximum temperature *T* is recorded as a function of time. As shown in Fig. 6 by means of an exemplary temperature signal, the temperature progression during the machining operation is irregular and therefore can be separated into two different phases. The first phase describes the run-in period, where the wire initially penetrates the CFRP material resulting in short-term peak values of *T*. Shortly after, the temperature value slightly decreases and a temperature equilibrium is reached, which represents the second phase. Since not the wire run-in characteristic but the cutting process itself is focused in this work, the run-in period is neglected for the temperature analysis. Instead, the recorded temperature *T* signal is averaged over the last 20 s of the cutting operation.

#### Roughness and waviness:

Roughness and waviness measurements are conducted using a Taylor Hobson Form Talysurf tactile profilometer according to standards DIN EN ISO 4287:2010 and DIN EN ISO 4288:1997. A cut-off filter of *λ*_*f*_ = 20 mm is arbitrarily chosen to remove long wavelength form deviation. In accordance with DIN EN ISO 4288:1997, the cut-off length *λ*_*c*_ is selected due to the expected *R*_*t*_, *R*_*z*_, or *R*_*a*_ values for non-periodic surface profiles. A cut-off filter of *λ*_*c*_ = 2.5 mm is applied to all profiles in order to separate roughness from waviness independent of the actual mean roughness measured to assure comparability between the specimens.

**Fig. 7:**
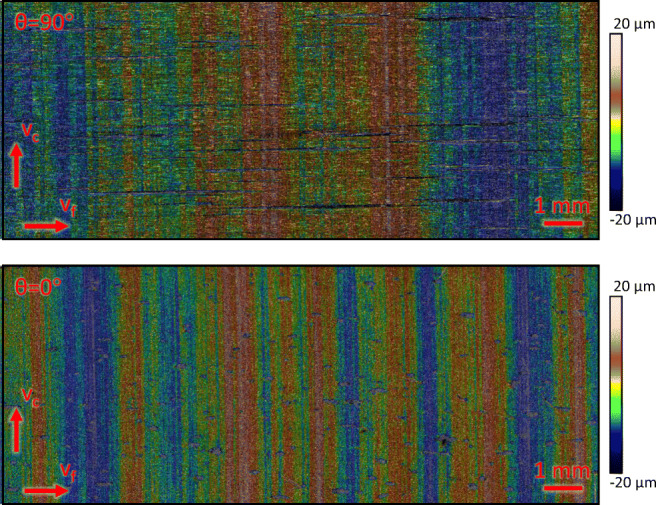
Microscopic images of the machined surface for *𝜃* = 90^∘^ and *𝜃* = 0^∘^, (*GD* = 50*%*, *v*_*f*_ = 100 *mm*/*min*, *v*_*c*_ = 25 *mm*/*min*, *t* = 7 *mm*); voids are seen as black areas between fibres and especially visible for *𝜃* = 90^∘^ in the top image

**Fig. 8:**
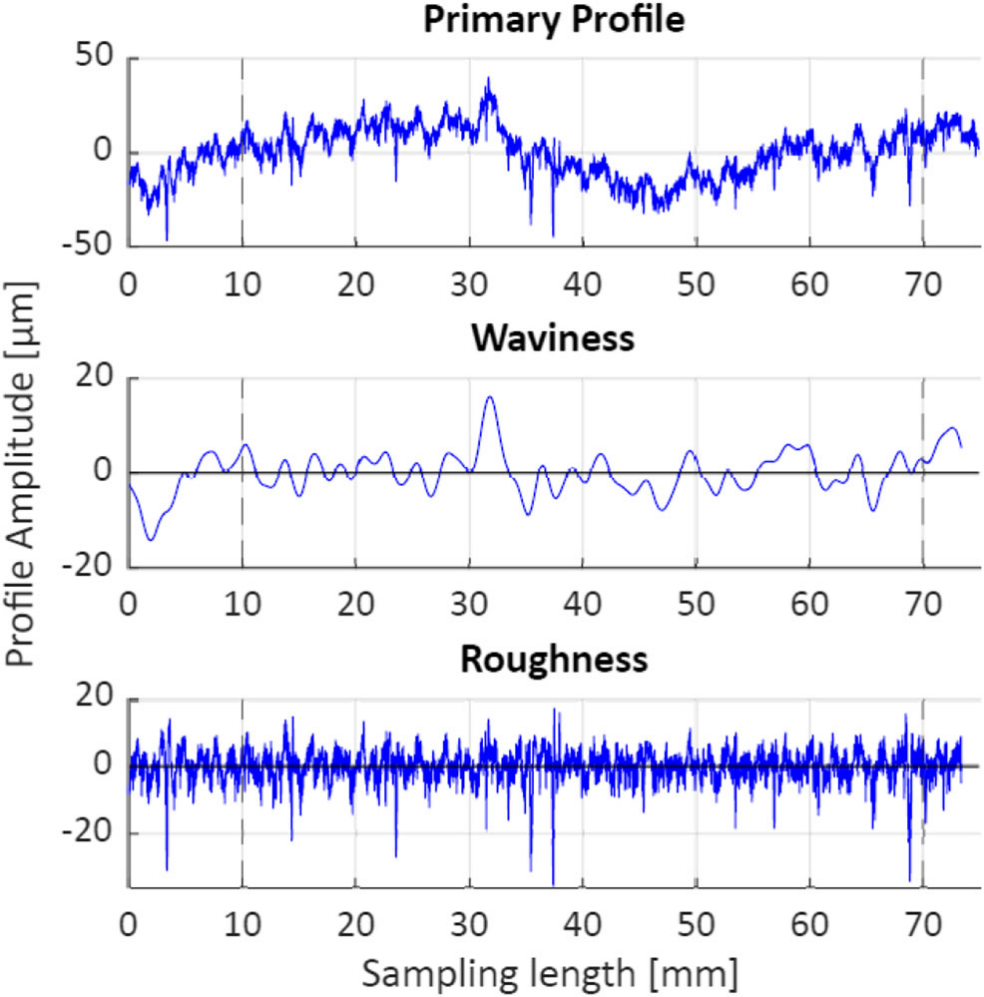
Example of a topography record, parameter setting 3 in Table 2, with vertical lines indicating the evaluated section; voids are visible as peaks in the magnitude of –30 *μm* in the roughness profile and the primary profile accordingly

DIN EN ISO 4288:1997 points out that the surface parameters are not suitable to describe the imperfection of surfaces. As a result, defects like pores and scratches may not be present in the measurement section used for evaluating surface parameters. Fulfilling this requirement is not possible without manually selecting and excluding faults from the measured profile, as irregular voids are present in the probes as seen in the microscopic images in Fig. 7 and the measured profile in Fig. 8.

In order to avoid manual intervention in the measurement data, the evaluated section *l*_*n*_ = 60 mm is chosen significantly larger than five profile filter lengths as suggested by the standard. The surface parameters *R*_*a*_, *R*_*z*_, *W*_*a*_, and *W*_*z*_ are sensitive to the choice and location of sections of measurement. Choosing 24 sampling lengths (*l*_*n*_ = 24*l*_*c*_, *l*_*c*_ = *λ*_*c*_) to determine the roughness parameters results in lower average values and effectively compensates large deviations in one measurement section. The effect on the peak-to-valley parameters is less pronounced since they are derived from maximum values only. They are also lowered by the averaging effect; however, since potentially more extreme peaks are considered, the mean peak-to-valley parameter may be larger than when considering fewer measurement sections. The form cut-off filter of *λ*_*f*_ = 20 mm results in a sampling length of *l*_*f*_ = 20 mm and therefore three sampling lengths can be fit into the whole waviness evaluation section.

In total, *l*_*n*_ = 60 mm of the cut specimen are evaluated, measured in direction of feed, ending 5 mm from the end of the cut. The surface is analysed, where the process has reached a steady state as discussed above in the paragraph “Process Forces”, which corresponds to approximately the second half of the specimen cut. The profile line is chosen to lay in the centre of the specimen half way between wire entrance and wire exit.

## Results

In the following section, the experimental results for the process forces, the workpiece surface temperature at the wire entrance, the surface roughness, and the surface waviness are visualised. The graphs show a box-plot-like representation, where each box corresponds to a setting, where the middle line represents the mean value and the bottom and top of the box are defined by the minimum and maximum recorded value respectively. Outliers and single data points are plotted as dots. The graphs are grouped to show the data for different fibre orientations *𝜃*, feed speeds *v*_*f*_, and grain densities *GD* first (settings 1–16), followed by a second pair of graphs, where the variation of the cutting speed *v*_*c*_ (setting 1 and 17) and workpiece thickness *t* (setting 1, 18, and 19) are compared. An analysis and discussion of the effects of the different variations of processing parameters on forces, temperature, and surface topography follows in Section 4. A table with all data points can be found in the Appendix.

### Process forces

**Fig. 9:**
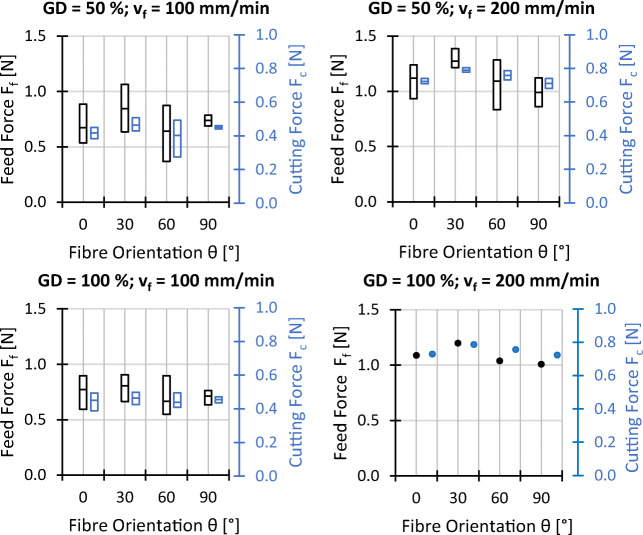
Experimental results process forces; *v*_*c*_ = 25 *m*/*s*, *t* = 7 *mm*, and all other settings as indicated

**Fig. 10:**
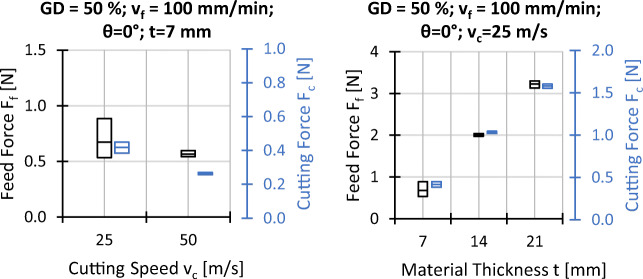
Experimental results process forces; *𝜃* = 0^∘^, *GD* = 50*%*, *v*_*f*_ = 100 *mm*/*min*

The evaluated process forces for the conducted experiments are shown in Figs. 9 and 10 respectively. The cutting forces are lower than the feed forces. The ratio of cutting to feed force varies with different sets of processing parameters. The fibre orientation impacts the process forces moderately.

According to Fig. 9, the feed force slightly increases if the fibre orientation changes from *𝜃* = 0^∘^ to *𝜃* = 30^∘^, but subsequently decreases again if the fibre orientation is further changed to *𝜃* = 60^∘^ and *𝜃* = 90^∘^. For the cutting force, a comparable trend is identified, however, less distinctive compared to the feed forces if the extreme values are taken into account. The influence of the grain density on both force components is nearly negligible. In contrast, the feed speed is identified as an important factor since both, the cutting and feed forces, rise significantly if the feed speed is increased from *v*_*f*_ = 100 mm/min to *v*_*f*_ = 200 mm/min.

According to Fig. 10, for *𝜃* = 0^∘^, increasing the cutting speed from *v*_*c*_ = 25 m/s to *v*_*c*_ = 50 m/s results in an overall reduction of the process forces as well as its fluctuations which, however, is more pronounced for the cutting force. For the variation of the material thickness, a nearly linear trend is observed, where the cutting and feed forces increases with increasing material thickness.

### Workpiece surface temperatures at the wire entrance

**Fig. 11:**
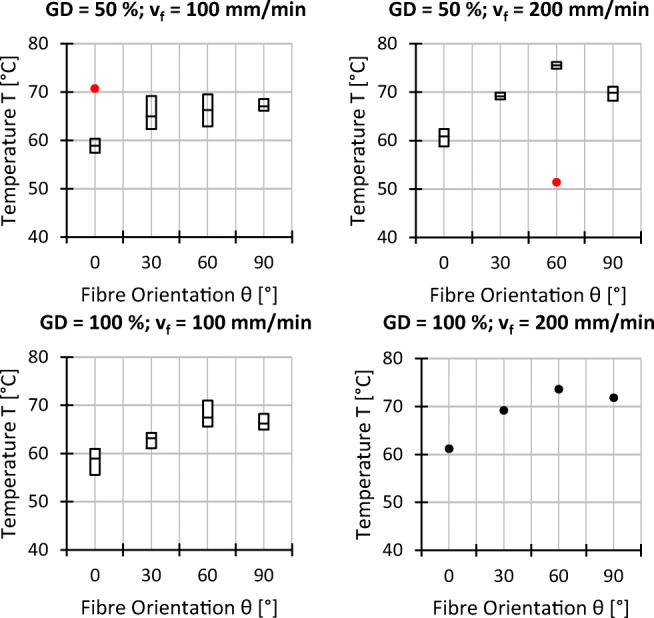
Experimental results workpiece temperature on the wire entrance side; *v*_*c*_ = 25 *m*/*s*, *t* = 7 *mm*, and all other settings as indicated

**Fig. 12:**
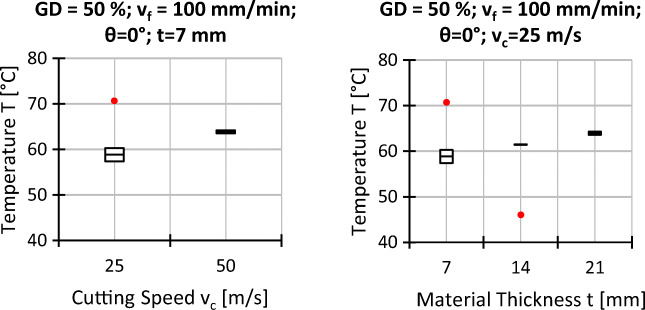
Experimental results workpiece temperature on the wire entrance side; *𝜃* = 0^∘^, *GD* = 50*%*, *v*_*f*_ = 100 *mm*/*min*

The workpiece surface temperatures at the wire entrance are shown in Figs. 11 and 12. It is found that the temperature is mainly affected by the fibre orientation, the feed and cutting speeds, and the material thickness while the influence of the grain density is nearly negligible.

According to Fig. 11, the surface temperature increases if the fibre orientations change from *𝜃* = 0^∘^ to *𝜃* = 30^∘^ and *𝜃* = 60^∘^. If the feed speed is increased, the dependency of the temperature on the fibre orientation remains; however, the absolute values are higher. Starting from *𝜃* = 60^∘^, with the exception of the parameter set with *GD* = 50*%* and *v*_*f*_ = 100 mm/min, the temperature values slightly decrease again if the fibre orientation is further increased to *𝜃* = 90^∘^. As shown in Fig. 12, increasing the cutting speed or the material thickness results in higher surface temperatures for *𝜃* = 0^∘^.

### Surface roughness and surface waviness

**Fig. 13:**
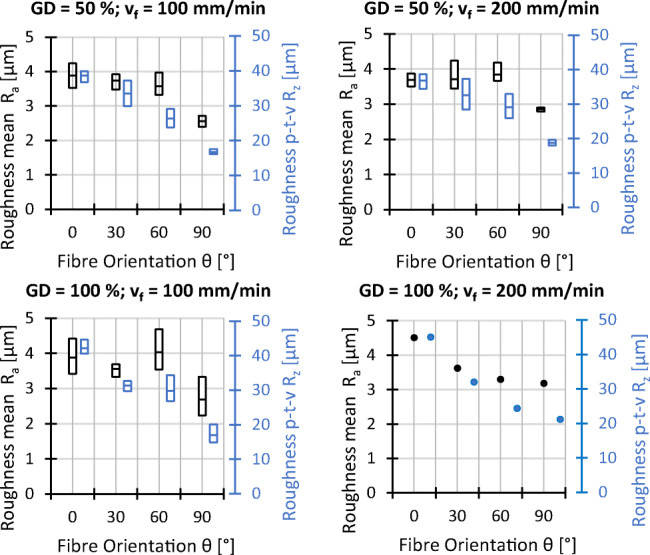
Experimental results roughness; *v*_*c*_ = 25 *m*/*s*, *t* = 7 *mm*, and all other settings as indicated

**Fig. 14:**
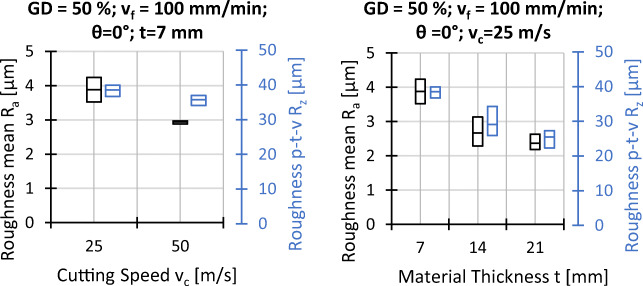
Experimental results roughness; *𝜃* = 0^∘^, *GD* = 50*%*, *v*_*f*_ = 100 *mm*/*min*

The surface roughness is evaluated in terms of the arithmetic average of the absolute values of the roughness profile *R*_*a*_ and average maximum profile height or peak-to-valley height *R*_*z*_, represented in Figs. 13 and 14. Both values are correlated and behave similarly with changes of processing parameters, where extremes have a much greater influence on *R*_*z*_ leading to a larger spread of measured values, as shown in both figures. With value ranges of 2.4 μm < *R*_*a*_ < 3.4 μm and 14.8 μm < *R*_*z*_ < 20.2 μm respectively, the lowest roughness is found for the fibre orientation of *𝜃* = 90^∘^, where the fibres are cut along their longitudinal axis. In addition, the roughness increases slightly with higher grain density and decreases with higher cutting speed and workpiece thickness.

**Fig. 15:**
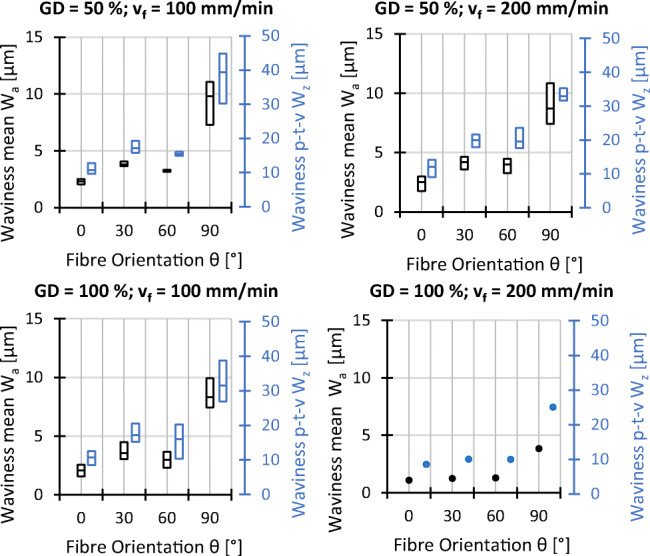
Experimental results waviness; *v*_*c*_ = 25 *m*/*s*, *t* = 7 *mm*, and all other settings as indicated

**Fig. 16:**
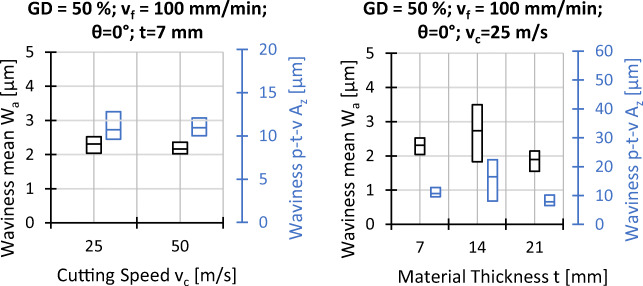
Experimental results waviness; *𝜃* = 0^∘^, *GD* = 50*%*, *v*_*f*_ = 100 *mm*/*min*

Analogous to the explanations before, *W*_*a*_ and *W*_*z*_ are characterised by different averaging methods so that *W*_*z*_ is more sensible to peak values. In this context, the largest profile height *W*_*z*_ is more than twice as large as the mean waviness *W*_*a*_ for all settings. In addition to this fact, the surface waviness shows a higher overall scatter between repetitions, which is shown in Figs. 15 and 16. Furthermore, the dependence on the fibre orientation is even more pronounced than that on the roughness. Compared to the surface roughness, the smallest surface waviness is found at *𝜃* = 0^∘^ and the largest at *𝜃* = 90^∘^. The cutting speed, however, does not seem to be of importance when changing from *v*_*c*_ = 25 m/s to *v*_*c*_ = 50 m/s and no clear trend for changes in feed speed and material thickness is observed.

### Qualitative surface evaluation

**Fig. 17:**
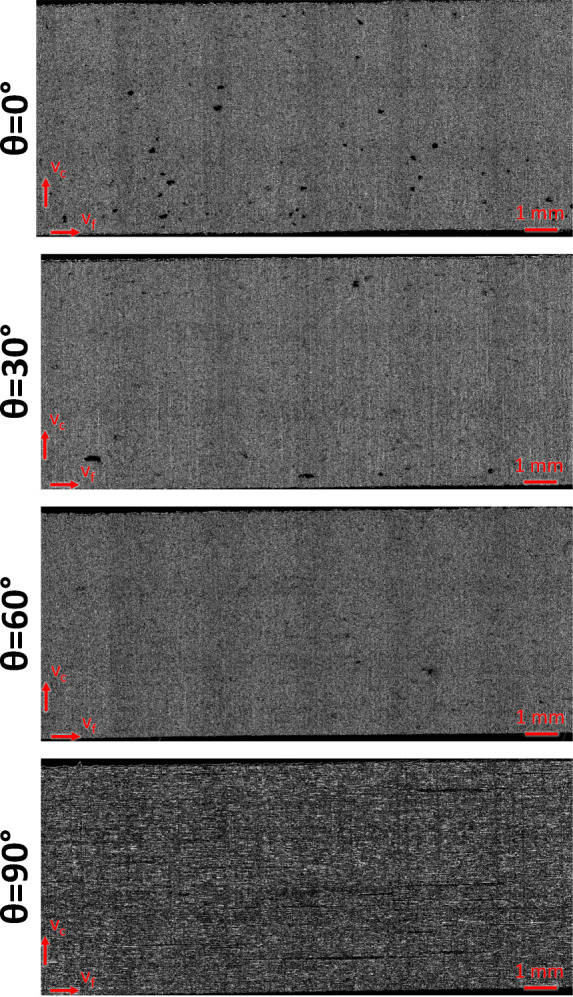
Microscopic images of wire sawn surfaces with respect to the fibre orientations *𝜃* = 0^∘^, *𝜃* = 30^∘^, *𝜃* = 60^∘^, *𝜃* = 90^∘^

In order to get a comprehensive understanding of the machined surface topography, a qualitative evaluation of the surface with respect to the fibre orientation is presented in Fig. 17. First and foremost, *𝜃* = 0^∘^ shows the most pores, followed by *𝜃* = 30^∘^ and *𝜃* = 60^∘^. For these fibre orientations, the largest identified pores have a length in the range of 0.5 mm. It is assumed that these pores already existed in the original material prior to the machining operation. In comparison, *𝜃* = 90^∘^ shows clearly longer pores, which are oriented along the carbon fibres with a maximum length of about 6 mm. Analogous to the previous fibre orientations, these pores are expected to have their origin in the material production. Generally, no fibre cracks are identified in the machined surface and the cutting edges are free of delamination and uncut fibres.

## Discussion

### Effects of changes of process parameters

**Fig. 18:**
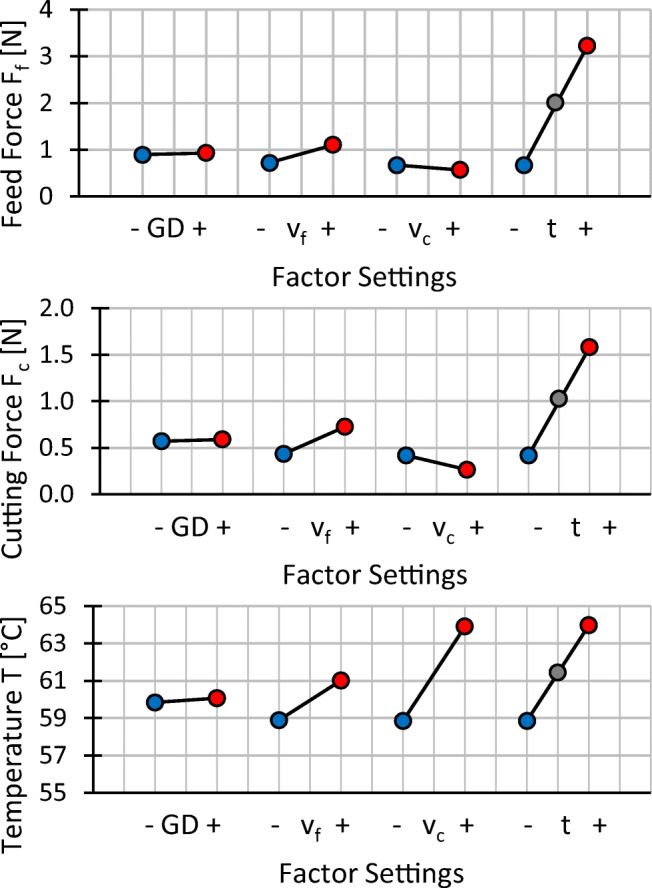
Influence of a change of a low to a high setting for all factors on feed force, cutting force and temperature

**Fig. 19:**
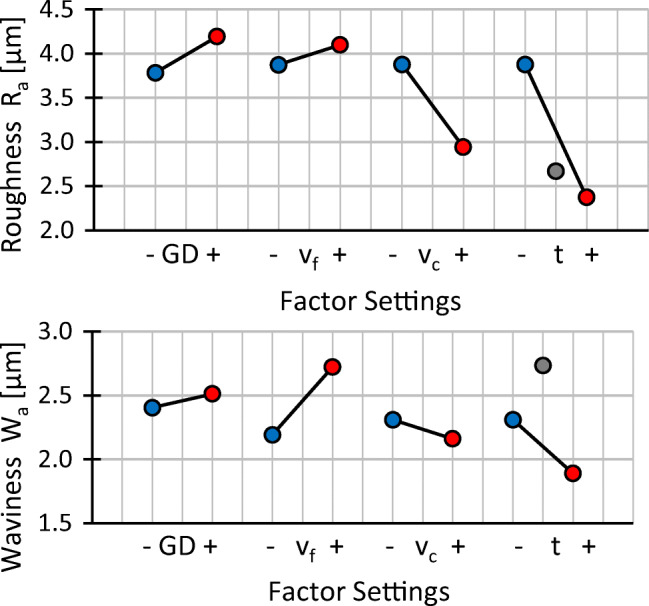
Influence of a change of a low to a high setting for all factors on the surface quality

Classic calculation of effects as known from the analysis of variance (ANOVA) is not possible with the experimental plan chosen, as the factors cutting speed and workpiece thickness are tested for one setting of grain density and feed speed only. A comparison of mean effects of changes in process parameter settings provides valuable insight nonetheless and is shown in Fig. 18 for the process forces and the temperature and in Fig. 19 for the surface quality. It should be noted that effects of changes in grain density are averaged for high and low settings of feed speed and effects of changes in feed speed are averaged for high and low settings of grain density respectively. Simultaneously, the effects of changes in cutting speed and workpiece thickness are derived from measurement points directly.

Increasing the grain density of the wires has no significant effect on the process forces and the considered workpiece surface temperature. An increase in feed speed leads to higher process forces and temperatures. Doubling the cutting speed from *v*_*c*_ = 25 m/s to *v*_*c*_ = 50 m/s has the same effect on the temperature as increasing the workpiece thickness three folds from 7 to 21 mm, whereas process forces decrease slightly. A wider workpiece correlates with strictly increasing feed and cutting force and has the most dominant effect observed in this comparison.

Due to the similar trends observed in Figs. 13 and 15 for the mean and maximum profile height for the surface roughness as well as for the surface waviness, the effect plots in Fig. 19 show the arithmetic mean deviations of the assessed profile for roughness *R*_*a*_ and waviness *W*_*a*_ only. Increasing grain density and feed speed leads to a higher roughness and waviness, while increased cutting speed leads to potentially lower values of *R*_*a*_ and *W*_*a*_. The medium settings of the workpiece thickness (grey dots) do not align with the falling trends of *R*_*a*_ and *W*_*a*_ in thinner workpieces. Since large scatter of the measurement results for waviness is observed for *t* = 14 mm in Fig. 16, the misalignment might be due to outliers, however the data does not permit a more detailed interpretation.

### Correlation between process parameters and surface quality

Changes in process parameters that increase temperatures and forces do not necessarily deteriorate the surface quality. Process temperature is not important as long as the matrix material is not damaged. Lowering process temperatures without sacrificing productivity is only possible by cooling actively as feed and cutting speed are both positively correlated with productivity and temperature. Cutting of CRFP often requires a dry process in order avoid material damages, meaning that cooling is only possible with air or another gas. This in turn is critical because of the fine, respirable, and cancerous dust that is created in the cutting operation. Few saws are able to accelerate a wire to *v*_*c*_ = 50 m/s and at that speed, temperatures are not critical. However, process temperature may limit the productivity on fast saws with thick workpieces.

Considering surface quality in terms of roughness and waviness, it appears that the roughness and waviness can be decreased by increasing the cutting speed and the workpiece thickness. Increasing the cutting speed leads to more grains passing the surface and removing peaks, thicker workpieces dampen wire vibrations more efficiently which may explain the trend for the roughness. Higher feed speed in turn decreases the amount of grains passing a surface and increases roughness. Higher grain density, which also leads to more grains passing the surface, however, this does not lead to a decreased surface roughness. It is possible that a higher grain density does not lead to an increase in kinematically active cutting edges for this kind of wire, impeding the expected effect. Waviness is the result of the wire drifting out of the cutting plane while it is effectively pulled back by the wire tension. With increasing feed and decreasing cutting speed, the wire has less time to cut back into the cutting plane, leading to a higher waviness with increasing feed speed and decreasing cutting speed. Based on the present data, a correlation between the waviness and the workpiece thickness cannot be deduced; a causal relationship would also not be immediately obvious.

### Correlation between fibre orientation and surface quality

**Fig. 20:**
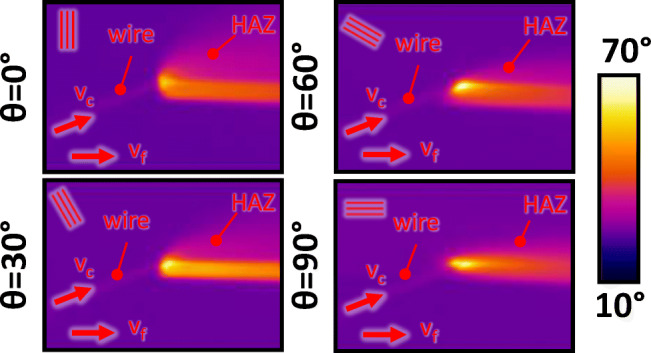
Heat affected zone in wire sawing UD CFRP with respect to the fibre orientation (*GD* = 100*%*, *v*_*f*_ = 200 *mm*/*min*, *v*_*c*_ = 25 *m*/*s*, *t* = 7 *mm*)

As previously shown in Fig. 11, the maximum surface temperature at the wire entrance is clearly affected by the fibre orientation of the CFRP material. In this context, Fig. 20 shows snapshots of the resulting HAZ as function of the fibre orientation.

Although this visualisation is shown with the example of one specific parameter set (*GD* = 100*%*, $v_{f} = 200~\text {mm}/\min \limits $, *v*_*c*_ = 25 m/s, *t* = 7 mm), it is representative for the remaining combinations of processing parameters. Obviously, the dimensions of the HAZ depend on the fibre orientations which are added in Fig. 20 as additional lines. The HAZ is the largest for *𝜃* = 0^∘^ and subsequently decreases if the fibre orientation angle is increased to *𝜃* = 30^∘^, *𝜃* = 60^∘^, and *𝜃* = 90^∘^. This is explained by the fact that heat conduction is much stronger along the carbon fibre than through the matrix material due to their differences in thermal conductivity. However, this means that for larger fibre orientation angles, the heat conduction to the surrounding CFRP material becomes worse since the fibres are more and more aligned with the feed direction of the wire. As a result of the impeded heat transport away from its origin in the cutting region, the maximum surface temperature at the wire entrance increases for larger fibre orientations which corresponds to the experimental findings presented in Section 3. Figure 20 also shows that heat is distributed into the bulk material while it is accumulated in the volumetrically smaller separated workpiece.

In terms of roughness and waviness, the influence of the fibre orientation is visible in Fig. 15, showing that the roughness and the waviness parameters vary only slightly for orientations *𝜃* = 0^∘^, *𝜃* = 30^∘^, and *𝜃* = 60^∘^, but are much larger for *𝜃* = 90^∘^. This circumstance can be explained with the fact that the wire has to cut the fibres along their axis for the *𝜃* = 90^∘^ orientation, which appears to significantly affect lateral wire deflection and furthermore leads to a different optical appearance of the surface as can be seen in Fig. 17.

### Wear

**Fig. 21:**
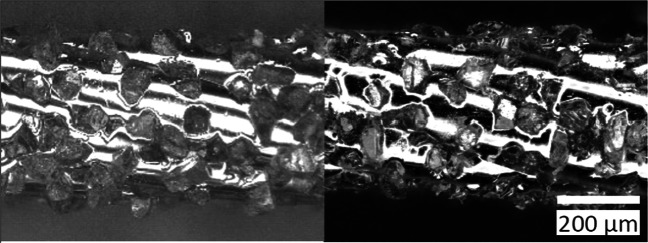
Wire with *GD* = 50*%* before (left) and after (right) cutting experiments

Wear, evaluated in terms of change of grain protrusion by determining the diameter of the envelope curve over the wire before and after the experiments, was not measurable. The dominant wear mechanism when cutting silicon with diamond wire is blunting of grains [16], which results in a decrease of protrusion and grain volume. The rounding off of cutting edges cannot be reliably quantified with the envelope curve or identified in microscopic images, see Fig. 21. Since no difference is observed, wear appears to be very small, which was to be expected based on the comparably small material volume removed per wire length.

Nonetheless, effects of wear are likely visible upon observation of process forces and temperatures within repetitions of the same parameter sets: With few exceptions, the forces and temperatures increase with increasing numbers of repetitions. Accordingly, the smallest force value is measured in the first repetition and the largest in the last repetition. This observation does not hold for roughness and waviness, where no trends with repetitions are noticed. All data is provided in Table 3 in the Appendix.

## Conclusion

In the scope of this experimental study, UD CFRP material with different material thicknesses is trimmed by a a single loop wire saw with diamond grains as fixed abrasives. In total, four different fibre orientations (*𝜃* = 0^∘^, *𝜃* = 30^∘^, *𝜃* = 60^∘^, *𝜃* = 90^∘^), two feed speeds ($v_{f} = 100~\text {mm}/\min \limits $, $v_{f} = 200~\text {mm}/\min \limits $), and two grain densities (*GD* = 50*%*, *GD* = 100*%*) are tested with a full factorial design of experiments while the cutting speed and the material thickness are kept constant with $v_{c} = 25~\text {mm}/\min \limits $ and *t* = 7 mm respectively. Furthermore, the influence of an increased cutting speed of $v_{c} = 50~\text {mm}/\min \limits $ and two thicker workpiece dimensions of *t* = 14 mm and *t* = 21 mm are tested while simultaneously, the remaining process parameters are fixed (*𝜃* = 0^∘^, *GD* = 50*%*, $v_{f} = 100~\text {mm}/\min \limits $).

In comparison with the results published by Zhang and Tani [33], this study confirms an increase of process forces with increasing feed speed and decreasing cutting speed. As these processing parameters determine the depth of cut per cutting edge and forces increase with increasing depth of cut [17, 29, 30], these results were to be expected. The study further confirms increasing roughness with increasing feed speed [3, 33] and decreasing cutting speed [3]. The lower significance of grain density on process forces has been observed by Pala et al. [17] for cutting silicon. The comparison shows that the process characteristics for cutting CFRP are similar to cutting mono-crystalline silicon.

This experimental study shows that diamond wire sawing is a suitable method for trimming of CFRP with respect to the achievable surface quality (small HAZ, comparable small surface roughness and waviness and no fibres along the cutting edges) and the machining efficiency (feed speed up to $v_{f} = 200~\text {mm}/\min \limits $).

## Data Availability

The raw data can be made available upon request. Processed data is available in Appendix, Table 3.

## Appendix: Experimental data

**Table 3:** Data of experiments

Setting	ID	*v*_*c*_ [$\frac {m}{s}$]	*v*_*f*_ [$\frac {mm}{min}$]	*GD* [*%*]	*𝜃* [^∘^]	*t* [*mm*]	*R*_*a*_ [*μ* m]	*R*_*z*_ [*μ* m]	*W*_*a*_ [*μ* m]	*W*_*z*_ [*μ* m]	*F*_*c*_ [*N*]	*F*_*f*_ [*N*]	*T* [^∘^C]
01a	V_02	25	100	50	0	7	4.24	39.88	2.37	9.68	0.42	0.53	70.7
01b	V_06	25	100	50	0	7	3.88	39.03	2.04	9.63	0.38	0.60	57.4
01c	V_22	25	100	50	0	7	3.52	36.70	2.52	12.81	0.45	0.89	60.3
02a	V_08	25	100	50	30	7	3.92	37.02	3.67	16.18	0.43	0.83	63.4
02b	V_09	25	100	50	30	7	3.81	33.49	4.05	19.33	0.45	0.63	62.3
02c	V_24	25	100	50	30	7	3.48	29.77	3.69	15.76	0.51	1.06	69.2
03a	V_01	25	100	50	60	7	3.32	23.78	3.31	14.87	0.28	0.37	62.9
03b	V_07	25	100	50	60	7	3.97	29.06	3.17	15.05	0.44	0.68	66.4
03c	V_20	25	100	50	60	7	3.44	26.09	3.16	16.10	0.49	0.87	69.5
04a	V_10	25	100	50	90	7	2.39	15.98	11.01	44.87	0.44	0.69	66.1
04b	V_12	25	100	50	90	7	2.56	16.23	11.06	43.06	0.45	0.74	66.5
04c	V_17	25	100	50	90	7	2.72	17.57	7.29	30.23	0.46	0.79	68.5
05a	V_04	25	200	50	0	7	3.50	34.41	1.77	9.03	0.71	0.93	58.8
05b	V_14	25	200	50	0	7	3.87	38.65	2.98	14.14	0.72	1.18	61.3
05c	V_18	25	200	50	0	7	3.69	37.30	2.75	12.97	0.74	1.24	62.4
06a	V_15	25	200	50	30	7	3.46	32.08	4.65	21.67	0.79	1.21	68.5
06b	V_16	25	200	50	30	7	3.44	28.37	4.32	20.31	0.78	1.22	69
06c	V_23	25	200	50	30	7	4.24	37.35	3.59	17.86	0.80	1.39	69.9
07a	V_03	25	200	50	60	7	4.19	32.93	3.26	17.55	0.73	0.83	51.4
07b	V_13	25	200	50	60	7	3.66	25.87	4.29	17.65	0.76	1.16	74.8
07c	V_21	25	200	50	60	7	3.67	28.37	4.46	23.56	0.79	1.28	76.2
08a	V_05	25	200	50	90	7	2.91	19.66	7.41	31.61	0.68	0.86	68.2
08b	V_11	25	200	50	90	7	2.88	18.58	7.89	31.86	0.71	0.99	70.2
08c	V_19	25	200	50	90	7	2.79	18.00	10.83	35.30	0.74	1.12	71.1
09a	V_28	25	100	100	0	7	4.42	44.68	1.56	8.49	0.39	0.59	55.6
09b	V_34	25	100	100	0	7	3.79	40.58	2.10	10.99	0.47	0.83	60.2
09c	V_38	25	100	100	0	7	3.41	41.07	2.57	12.57	0.49	0.90	61
10a	V_27	25	100	100	30	7	3.66	31.83	3.06	15.22	0.43	0.66	61.1
10b	V_31	25	100	100	30	7	3.32	29.67	4.49	20.49	0.46	0.85	64.2
10c	V_40	25	100	100	30	7	3.69	32.64	3.06	15.91	0.50	0.91	64.3
11a	V_25	25	100	100	60	7	4.68	34.39	2.31	10.30	0.41	0.55	65.7
11b	V_26	25	100	100	60	7	3.87	26.83	2.96	17.35	0.41	0.55	65.6
11c	V_39	25	100	100	60	7	3.54	28.13	3.66	20.27	0.49	0.90	71
12a	V_30	25	100	100	90	7	3.34	20.05	9.93	38.80	0.44	0.63	65
12b	V_33	25	100	100	90	7	2.22	14.86	7.59	26.85	0.47	0.74	65.5
12c	V_37	25	100	100	90	7	2.50	15.88	7.44	28.94	0.46	0.76	68.2
13a	V_29	25	200	100	0	7	4.51	44.98	2.95	13.66	0.73	1.09	61.2
14a	V_36	25	200	100	30	7	3.62	32.01	3.34	16.02	0.79	1.20	69.2
15a	V_32	25	200	100	60	7	3.30	24.34	3.47	15.92	0.76	1.04	73.6
16a	V_35	25	200	100	90	7	3.18	21.18	10.27	40.07	0.72	1.01	71.8
17a	V_41	50	100	50	0	7	2.97	36.97	2.36	12.07	0.26	0.56	64.2
17b	V_42	50	100	50	0	7	2.97	36.07	2.10	10.76	0.27	0.54	63.5
17c	V_43	50	100	50	0	7	2.88	34.08	2.02	10.03	0.27	0.60	64
18a	V_44	25	100	50	0	14	2.29	25.87	3.49	22.42	1.02	1.97	46
18b	V_45	25	100	50	0	14	3.13	34.31	2.89	18.93	1.02	2.02	61.4
18c	V_46	25	100	50	0	14	2.58	26.92	1.82	8.15	1.05	2.04	61.5
19a	V_47	25	100	50	0	21	2.19	22.30	1.55	6.50	1.55	3.13	63.6
19b	V_48	25	100	50	0	21	2.63	27.31	2.14	10.18	1.58	3.24	64
19c	V_49	25	100	50	0	21	2.30	26.86	1.98	6.90	1.60	3.30	64.3
